# Exposure to Mycotoxin-Mixtures via Breast Milk: An Ultra-Sensitive LC-MS/MS Biomonitoring Approach

**DOI:** 10.3389/fchem.2020.00423

**Published:** 2020-05-19

**Authors:** Dominik Braun, Chibundu N. Ezekiel, Doris Marko, Benedikt Warth

**Affiliations:** ^1^Department of Food Chemistry and Toxicology, Faculty of Chemistry, University of Vienna, Vienna, Austria; ^2^Department of Microbiology, Babcock University, Ilishan Remo, Nigeria

**Keywords:** exposure assessment, food safety, exposome, infant and public health, environmental contaminants

## Abstract

Exposure to natural food contaminants during infancy may influence health consequences later in life. Hence, breast milk may serve as a vehicle to transport these contaminants, including mycotoxins, from mothers to their infants. Analytical methods mostly focused on single exposures in the past, thus neglecting co-occurrences and mixture effects. Here, we present a highly sensitive multi-biomarker approach by a sophisticated combination of steps during sample preparation including a Quick, Easy, Cheap, Effective, Rugged and Safe (QuEChERS) extraction followed by a solid phase extraction (SPE) cleanup and utilizing stable isotopes for compensating challenging matrix effects. The assay was validated in-house, reaching limits of detection (LOD) for all 34 analytes in the range of 0.1 to 300 ng/L with satisfying extraction efficiencies (75–109%) and stable intermediate precisions (1–18%) for most analytes. Compared to a similar multi-mycotoxin assay for breast milk, LOD values were decreased by a factor of 2–60x enabling the assessment of chronic low-dose exposures. The new method was applied to a small set of Nigerian breast milk samples (*n* = 3) to compare results with already published data. Concentration levels of samples that were found to be contaminated before could be confirmed. In addition, other mycotoxins were determined in all three samples, for example the newly investigated alternariol monomethyl ether (AME) was found for the first time in this biological fluid at concentrations up to 25 ng/L. Moreover, in a pooled Austrian sample obtained from a milk bank, trace amounts of multiple mycotoxins including AME (1.9 ng/L), beauvericin (5.4 ng/L), enniatin B (4.7 ng/L), enniatin B_1_ (<LOQ), ochratoxin A (<LOQ) and the estrogenic zearalenone (<LOQ) confirmed co-occurrence and exposure even in a country with high food safety standards. In conclusion, the method facilitates the determination of mycotoxins at ultra-trace levels in breast milk, enabling the generation of occurrence data necessary for comprehensive co-exposure assessment.

## Introduction

The benefits of breast milk for infants concerning gastrointestinal function, lower risk of infectious diseases or the development of the immune system have been well-documented (Horta et al., 2007). Positive effects of breastfeeding for the mother are associated with emotional bonding, reduced risk for the development of type 2 diabetes or breast cancer (Palmer et al., 2014). However, mothers are likely exposed to food contaminants such as mycotoxins due to exposure via naturally contaminated foodstuffs. These toxins may be transferred to infants via breast milk. The exposure of infants is critical because they are generally more susceptible, particularly premature newborns, and have a less developed immune system during the first months of life (EFSA Scientific Committee et al., 2017).

Mycotoxins are toxic secondary metabolites produced by a variety of fungi, including *Aspergillus, Penicillium*, and *Fusarium* species. Harmful effects were previously reported in many animal studies involving immune suppression, target organ toxicity or the development of cancer (Bondy and Pestka, 2000; IARC, 2002; CAST, 2003). Aflatoxins are associated with suppressed immune functions and impaired growth of children (Turner et al., 2003; Gong et al., 2004, 2012). Ochratoxin A (OTA) is known as a nephrotoxic agent in several animal species, due to its accumulation in the kidney (Malir et al., 2013). Trichothecenes are most prevalently produced by *Fusarium* toxins and are known for their emetic effects and their suppression of immune functions [EFSA Panel on Contaminants in the Food Chain (CONTAM) et al., 2017]. Zearalenone (ZEN) is commonly found in cereals in different world regions and interferes with the endocrine system, due to its high affinity to the estrogen receptor (Kowalska et al., 2018). The toxicity of emerging mycotoxins such as the *Alternaria* toxins alternariol (AOH), its monomethyl ether (AME) or tentoxin (TEN) has not been fully assessed or data are clearly lacking to classify these toxins. However, AOH and AME are known for their genotoxic effects *in vitro* by acting as topoisomerase I or II poison (Jarolim et al., 2017). Furthermore, recent studies indicate endocrine disruptive and immune modulatory properties (Dellafiora et al., 2018; Kollarova et al., 2018; Schmutz et al., 2019).

Several reports are available on the occurrence of mycotoxins in breast milk and were reviewed before by Warth et al. (2016) and Sengling Cebin Coppa et al. (2019). Briefly, aflatoxin M_1_ (AFM_1_) and OTA are the main mycotoxins which were assessed utilizing mostly enzyme-linked immunosorbent assay or liquid chromatography coupled to fluorescence detection in different world regions and with a high variance in occurrence. While these published methods lack the specificity of a targeted LC-MS/MS approach, we developed and validated such a method recently to assess multiple classes of mycotoxins in breast milk (Braun et al., 2018). However, a pilot survey revealed that contamination levels were near or below the LOQ for all detected analytes. To enable the accurate quantification of low dose chronic early-life exposures and thoroughly evaluate their real-life impact, a highly sensitive approach has to be developed.

The aim of the present work was therefore to significantly improve the sensitivity of our previously developed LC-MS/MS methodology to assess mycotoxin exposures even in countries with high food safety standards. Consequently, the main focus was to optimize the sample preparation protocol and to compare sensitivity and other critical performance parameters using two triple quadrupole mass spectrometers of different vendors. Moreover, new mycotoxins of high toxic potential were included to collect data for a more detailed picture of co-exposure. The applicability of the significantly improved LC-MS/MS tool was subsequently demonstrated by analyzing a small set of randomly selected samples obtained from a Nigerian cohort and by examining a pooled Austrian breast milk sample.

## Materials and Methods

### Chemicals and Reagents

LC-MS grade solvents [water, acetonitrile (ACN) and methanol (MeOH)] were purchased from Honeywell (Seelze, Germany). Acetic acid, ammonium acetate, anhydrous magnesium sulfate, formic acid and sodium chloride were bought from Sigma-Aldrich (Vienna, Austria). The following mycotoxins reference standards were purchased: Aflatoxin B_1_ (AFB_1_), AFB_2_, AFG_1_, AFG_2_, deoxynivalenol (DON), OTA, nivalenol (NIV), sterigmatocystin (STC), fumonisin B_1_ (FB_1_), FB_2_, T-2 toxin, alpha zearalenol (α-ZEL), β-ZEL, alpha zearalanol (α-ZAL), β-ZAL, zearalanone (ZAN) and ZEN from RomerLabs (Tulln, Austria). Enniatin A (Enn A), Enn A_1_, Enn B, Enn B_1_, tenuazonic acid (TeA), and TEN from Sigma-Aldrich (Vienna, Austria). Aflatoxin metabolites AFM_1_, AFM_2_, AFP_1_, AFQ_1_, AFB_1_-N7-guanine adduct, as well as AME, AOH, beauvericin (BEA), citrinin (CIT), HT-2 toxin, ochratoxin alpha (OTα), ochratoxin B (OTB) from Toronto Research Chemicals (Ontario, Canada). Dihydrocitrinone (DH-CIT) was kindly provided by Prof. Michael Sulyok (IFA-Tulln, Austria). Solid reference materials were dissolved in ACN, except the fumonisins (ACN/H_2_O, 1/1, v/v) and AFB_1_-N7-guanine (ACN/H_2_O/acetic acid, 75/24/1, v/v/v) to reach individual stock solutions with final concentrations of 5-500 μg/mL which were stored at −20°C. Internal standards (IS) [^13^C]-AFM_1_, [^13^C]-CIT, [^13^C]-DON, [^13^C]-FB_1_, [^13^C]-NIV, [^13^C]-OTA, [^13^C]-ZEN were purchased from RomerLabs (Tulln, Austria). [^2^H]-AOH was kindly provided by Prof. Michael Rychlik (TU Munich, Germany). To prepare a multi-standard working solution containing all analytes, the stock solutions were diluted in MeOH reaching concentrations of 36–17,000 ng/mL. A fresh IS mixture was prepared containing the following concentrations: [^2^H]-AOH (4.5 ng/mL), [^13^C]-AFM_1_ (0.4 ng/mL), [^13^C]-CIT (0.1 ng/mL), [^13^C]-DON (4.0 ng/mL), [^13^C]-FB_1_ (4.0 ng/mL), [^13^C]-NIV (4.0 ng/mL), [^13^C]-OTA (4.5 ng/mL) and [^13^C]-ZEN (4.5 ng/mL).

### Breast Milk Samples

Breast milk samples from Austria were kindly provided by the Semmelweis Women's Clinic in Vienna, Austria. Samples of more than 150 women were collected in 2015, pooled and stored at −20°C. This pooled sample was used for method development and optimization in the current as well as in the previous published work (Braun et al., 2018). This study was permitted by the Ethic Committee of the University of Vienna (IRB#00157). For the Nigerian breast milk samples, randomly selected breast milk aliquots (*n* = 3) originating from mothers in Ogun state, Nigeria which were part of an earlier study (Braun et al., 2018), were re-extracted. Ethical approval was granted by the responsible Ethical Committee of Babcock University under the number: #BUHREC294/16.

### Sample Preparation Protocol

Several different clean-up and enrichment steps were evaluated, while the main extraction procedure was based on our established QuEChERS approach (Braun et al., 2018) to which an SPE clean-up and enrichment step was added. Briefly, the final protocol was as follows: 1 mL of human breast milk was vortexed, 1 mL of acidified ACN (1% formic acid) added and vigorously shaken for 3 min. Then, 0.4 g anhydrous magnesium sulfate and 0.1 g sodium chloride were separately added and mixed again (3 min). After centrifugation for 10 min (4,750 × g at 10°C) the upper layer (ACN, 950 μL) was transferred to a new micro-reaction tube and chilled at −20°C for 2 h. After a second centrifugation step (2 min at 14,000 × g, 4°C), 900 μL of the supernatant was directly transferred to a reservoir, which was preloaded with 17.1 mL water, onto an Oasis PRiME HLB® SPE column (Waters, Milford, MA). The SPE cartridge was equilibrated with 1 mL ACN, and 1 mL H_2_O/ACN (95/5, v/v) before the water containing reservoir was attached. After washing twice with 500 μL H_2_O/ACN (95/5, v/v), mycotoxins were eluted with three times 500 μL pure ACN. The extract was dried using a vacuum concentrator (Labconco, Missouri, USA), reconstituted in 81 μL MeOH/ACN (50:50, v:v) and fortified with 9 μL of the IS mixture, resulting in an overall concentration factor of 10 for the analytes of interest. Then, samples were vortexed, ultra-sonicated for 5 min and transferred to amber LC-vials containing a micro-insert. Subsequently, 3 μL were injected onto the LC-MS/MS system.

### LC-MS/MS Analysis

LC-MS chromatographic analysis of purified breast milk extracts was performed based on Braun et al. (2018). In short, chromatographic separation was achieved utilizing an Acquity HSS T3 column (1.8 μm, 2.1 × 100 mm) guarded with a VanGuard pre-column (1.8 μm, Waters, Vienna, Austria). The column oven was set to 40°C and the autosampler maintained at 10°C. Gradient elution was carried out using an acidified ammonium acetate solution in water (5 mM, acidified with 0.1% acetic acid; A) and MeOH (B). Two LC-MS/MS instruments were used for method performance comparison. First, a Dionex Ultimate 3000 UHPLC coupled to a TSQ Vantage triple quadrupole mass spectrometer (Thermo Scientific, Vienna, Austria) equipped with an electrospray ionization interface (ESI) was used. Second, the method was transferred to an Agilent 1290 Infinity II LC coupled to a Sciex QTrap6500^+^ (Darmstadt, Germany) mass spectrometer. The MS was equipped with a Turbo-V™ ESI source.

LC-MS/MS operation parameters are reported in Table S1 (TSQ Vantage) in the Supplementary Material (SM) and Table 1 (QTrap6500^+^) for both instruments, respectively. Ion source parameters were optimized for each instrument and are either described in the SM or elsewhere (Braun et al., 2018). The final instrument setup used consisted of the Agilent 1290 Infinity II coupled to the Sciex QTrap6500^+^ instrument. The Chromeleon™ Chromatography Data System (version 3) and Analyst (version 1.7) software were used for data acquisition and instrument control. Data evaluation was executed using either the Tracefinder™ (version 3.3) or MultiQuant (3.0.3) software package.

**Table 1:** Optimized analyte specific MS and MS/MS parameters utilized on the QTrap6500^+^ instrument.

**Analyte**	**${\text{t}}_{\text{R}}^{\text{a}}$**	**Precursor ion**	**Ion species**	**Product ion^b^**	**DP^c^**	**CE^d^**	**CXP^e^**	**Ion ratio^f^**
	**(min)**	**(*****m/z*****)**		**(*****m/z*****)**	**(V)**	**(V)**	**(V)**	**(%)**
Aflatoxicol	5.9	297.0	[M-H_2_O+H]^+^	269.1/115.0	71	29/83	12/14	98
Aflatoxin B_1_	5.2	313.0	[M+H]^+^	241.0/213.0/259.2	106	49/61/43	14/16/18	65
Aflatoxin B_2_	5.0	315.0	[M+H]^+^	243.0/203.0	125	53/49	16/12	46
Aflatoxin G_1_	4.7	329.1	[M+H]^+^	243.1/200.0/214.6	86	39/59/46	14/12/14	67
Aflatoxin G_2_	4.5	331.1	[M+H]^+^	313.2/245.2	111	35/43	18/14	59
Aflatoxin M_1_	4.5	329.1	[M+H]^+^	273.2/229.1	91	35/59	16/12	54
^13^C-Aflatoxin M_1_	4.5	346.0	[M+H]^+^	288.2	91	35	16	-
Aflatoxin M_2_	4.3	331.0	[M+H]^+^	285.2/259.0/241.0	96	33/33/57	14/16/14	99
Aflatoxin P_1_	4.8	299.1	[M+H]^+^	270.7/215.1/171.1	126	35/38/56	18/11/17	30
Aflatoxin Q_1_	4.4	328.7	[M+H]^+^	206.0/177.0	121	33/47	14/12	71
Aflatoxin B_1_-N7-guanine	4.0	480.0	[M+H]^+^	152.1/135.0	46	23/85	10/14	41
Alternariol	6.3	257.0	[M-H]^−^	215.0/213.0	−100	−36/−34	−11/−11	137
^2^H-Alternariol	6.3	261.0	[M-H]^−^	150.0	−110	−46	−5	-
Alternariol monomethyl ether	8.2	271.1	[M-H]^−^	256.0/227.0	−95	−32/−50	−13/−9	17
Beauvericin	11.0	801.5	[M+NH_4_]^+^	244.2/134.0/262.1	66	42/99/41	14/14/18	108
Citrinin	5.2	281.0	[M+MeOH-H]^−^	249.0/205.0	−50	−24/−33	−7/−7	56
^13^C-Citrinin	5.2	294.3	[M+MeOH-H]^−^	217.1	−40	−32	−17	-
Deoxynivalenol	3.1	355.1	[M+OAc]^−^	265.2/59.2	−70	−24/−40	−13/−8	940
^13^C-Deoxynivalenol	3.1	370.1	[M+OAc]^−^	278.8	−20	−22	−15	-
Dihydrocitrinone	4.5	265.0	[M-H]^−^	177.0/203.0/147.1	−25	−34/−40/−46	−11/−17/−15	23
Enniatin A	11.5	699.4	[M+NH_4_]^+^	210.1/100.1/228.0	106	43/91/47	12/12/18	69
Enniatin A_1_	11.3	685.4	[M+NH_4_]^+^	210.1/100.2/196.1	96	41/89/39	8/8/14	70
Enniatin B	10.9	657.5	[M+NH_4_]^+^	196.3/214.1	81	45/47	18/18	63
Enniatin B_1_	11.1	671.4	[M+NH_4_]^+^	196.0/210.0	111	43/41	12/12	70
Fumonisin B_1_	6.2	722.5	[M+H]^+^	334.4/352.3	121	57/55	4/12	95
^13^C-Fumonisin B_1_	6.2	756.3	[M+H]^+^	356.3	130	46	10	-
Fumonisin B_2_	7.9	706.5	[M+H]^+^	336.4/318.4	126	59/51	8/2	44
HT-2 toxin	6.2	442.2	[M+NH_4_]^+^	263.1/215.0	76	21/21	19/19	227
Nivalenol	2.7	371.1	[M+OAc]^−^	281.1/59.1	−75	−22/−42	−15/−7	92
^13^C-Nivalenol	2.7	386.0	[M+OAc]^−^	295.2	−75	−22	−15	-
Ochratoxin A	6.5	404.0	[M+H]^+^	239.0/102.0	91	37/105	16/14	34
^13^C-Ochratoxin A	6.5	424.0	[M+H]^+^	250.0	51	33	12	-
Ochratoxin B	5.5	370.1	[M+H]^+^	205.0/103.1	86	33/77	12/16	30
Ochratoxin α	4.4	254.9	[M-H]^−^	166.9/123.0/110.9	−90	−36/−40/−44	−11/−17/−21	21
Sterigmatocystin	8.1	325.1	[M+H]^+^	281.1/310.2/253.1	96	51/35/57	16/18/16	84
T-2 toxin	7.0	484.3	[M+NH_4_]^+^	215.2/185.1	56	29/31	18/11	88
Tentoxin	6.5	413.3	[M-H]^−^	141.0/271.1	−105	−30/−24	−11/−15	64
Zearalanone	7.5	319.1	[M-H]^−^	161.0/107.0/137.0	−125	−38/−40/−38	−15/−13/−17	64
α-Zearalanol	7.2	321.1	[M-H]^−^	277.1/235.1/161.0	−120	−30/−32/−38	−18/−17/−9	6.0
β-Zearalanol	6.4	321.1	[M-H]^−^	277.1/303.1	−120	−30/−30	−18/−20	29
Zearalenone	7.7	317.1	[M-H]^−^	175.0/131.1/160.0	−110	−34/−42/−40	−13/−8/−11	73
^13^C-Zearalenone	7.7	335.2	[M-H]^−^	185.1	−110	−34	−13	-
α-Zearalenol	7.4	319.2	[M-H]^−^	160.1/130.1	−115	−44/−50	−13/−20	61
β-Zearalenol	6.7	319.2	[M-H]^−^	160.1/130.1	−115	−44/−50	−13/−20	63

a *Retention time*.

b *Quantifier/qualifier/confirming ion*.

c *Declustering potential*.

d *Collision energy*.

e *Cell exit potential*.

f *Calculated as (qualifier/quantifier × 100) in matrix-matched standard*.

### Validation Experiments

Since certified reference materials for the analysis of mycotoxins in human breast milk are not commercially available, the optimization and validation of the presented method were performed as previously described by Braun et al. (2018). An Austrian pooled breast milk sample intended for method development and optimization was spiked with mycotoxin analytical reference standards and extracted following our new protocol. According to the European Commission Decision 2002/657/EC (EC 2002) and the EuraChem Laboratory Guide (Magnusson and Örnemark, 2014), concerning the performance of analytical methods and their validation, the following parameters were evaluated: sensitivity, selectivity, repeatability (intraday precision, RSD_r_), intermediate precision (interday precision, RSD_R_), linearity, extraction recovery (R_E_) and signal suppression or enhancement. Calibration standards were prepared in neat solvent and unspiked pooled breast milk extracts (matrix matched standards). Matrix-matched calibration curve (1/x weighted) for each mycotoxin was established using at least five concentration levels. In case of a natural contamination of the unspiked pooled breast milk extract, results reported were evaluated by standard addition method. For mycotoxins for which IS were available, peak area ratios were used for quantification, while for mycotoxins without IS all calculations were performed using the peak area. Limits of detection (LOD) and limits of quantification (LOQ) were calculated by dividing the standard deviation of the lowest spiking level with the square root of replicated experiments. This value was multiplied by a factor of three and six to obtain LOD and LOQ values, respectively (Magnusson and Örnemark, 2014; Braun et al., 2018).

## Results and Discussion

### Optimization of the Multi-analyte QuEChERS/SPE Extraction Procedure

Although detection of environmental contaminants utilizing LC-MS/MS is very sensitive and selective, complex biological matrices such as human breast milk may diminish this advantage by matrix effects during ESI. To resolve these drawbacks, an effective clean-up step is necessary to disrupt the matrix and efficiently extract all analytes of interest. The QuEChERS approach demonstrated excellent recovery of targeted analytes as shown before (Braun et al., 2018). This earlier developed sample clean-up approach was modified to detect ultra-trace levels of mycotoxins and chronic low-dose exposures to enable proper exposure assessments. A variety of different approaches were integrated to the earlier established QuEChERS approach, including dispersive to traditional SPE and thereafter tested.

**Figure 1:**
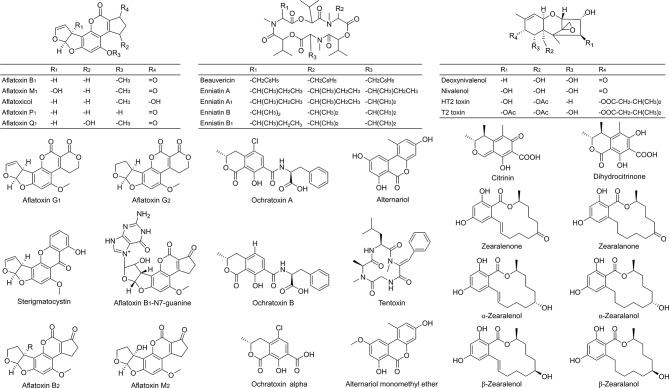
Chemical structures of the 34 investigated mycotoxins and some of their metabolites as included in the optimized LC-MS/MS method. Validation criteria were met for 29 analytes (excluding alternariol, aflatoxin B_1_-N7-guanine, deoxynivalenol, dihydrocitrinone and nivalenol).

**Figure 2:**
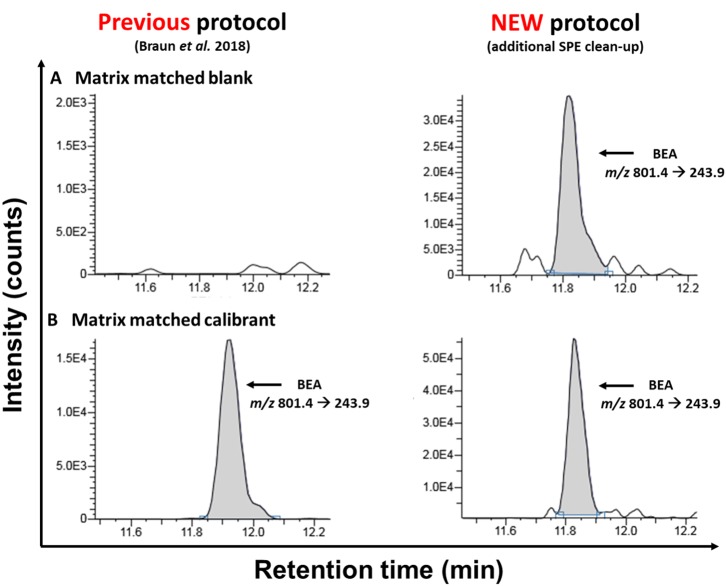
Comparison of MRM-chromatograms of matrix-matched “blank” samples **(A)** and matrix-matched calibrants **(B)** after the extraction with the old sample preparation protocol [according to Braun et al. (2018)] and the newly presented approach acquired on the TSQ Vantage instrument.

Dispersive SPE materials are known to bind free fatty acids and other co-extracted matrix components. These are frequently used in combination with the QuEChERS approach in pesticide analysis for extracting analytes from complex matrices (Lehmann et al., 2018). However, dispersive SPE, utilizing materials like C18, poly-secondary amine (PSA), zirconium salts (Z-Sep), or combinations thereof, had no significant improvement on any analyte. On the contrary, the Z-Sep material bound specifically CIT, DH-CIT and ochratoxins and decreased their extraction recovery (R_E_ <10%). Thus, the dispersive SPE approach was discarded. Hybrid-SPE technologies like phospholipid SPE materials bind proteins and phospholipids, however, analytes like OTA and CIT were selectively bound resulting in 1% extraction efficiency. Subsequently, a traditional SPE clean-up was investigated. Here, the integration of this step was a crucial factor to efficiently extract the targeted analytes. Hence, in the newly established protocol an evaporation step of ACN was avoided and the QuEChERS extract was directly diluted to 5% in a H_2_O preloaded SPE-reservoir (Figure S1). The operating conditions were thoroughly optimized to guarantee good extraction recoveries for the extremely diverse analytes (Figure 1). Moreover, the resulting eluate was concentrated by a factor of 10. The extraction efficiency and sensitivity of pre-experiments were highly satisfying for most toxins on the TSQ Vantage (Figure 2) and on the QTrap6500^+^ instrument. Consequently, this approach was selected for method validation.

### Method Transfer

The QTrap6500^+^ instrument was recently used to assess trace levels of mycotoxins in urine (Šarkanj et al., 2018). Thus, this instrument was selected to examine sensitivity and other critical performance parameters useful for assessing and quantifying mycotoxins in human breast milk. Here, a major advantage is the scheduled multiple reaction monitoring (MRM) data acquisition which allowed the reduction of the methods cycle time to 0.3 s by maintaining or even increasing the dwell time of nearly all MRM transitions ranging from 8 ms up to 80 ms. The basis to perform scheduled MRM analysis is stable chromatographic analyte retention as the transition is only measured in a time window near the expected retention time (Figures S2, S3). Instrument performance and method validation was examined using both triple quadrupole instruments. In the following sections, however, data obtained from the QTrap6500^+^ instrument setup are reported and discussed. MS and MS/MS parameters are reported in Table 1. This set-up clearly demonstrated significant improvement in performance and sensitivity compared to the TSQ Vantage instrument with lower LOD values by a factor of 3-5x on average (Tables 2, 3 and Table S2). Importantly, it needs to be highlighted that one of the instruments is state-of-the-art, while the other instrument is available on the market for more than 10 years. However, the MS and MS/MS specific parameters as well as validation experiments carried out on the TSQ Vantage instrument are summarized in Tables S1, S2 for the interested reader, as high-end state-of-the-art instruments are frequently not affordable for research labs.

**Table 2:** Performance characteristics of the method as obtained during in-house validation on the QTrap6500^+^ instrument including concentration range of matrix matched standard calibration, regression coefficient (*R*^2^), spiking levels, recoveries of the extraction step (R_E_), intermediate precision (RSD_R_), repeatability (RSD_r_), signal suppression/enhancement (SSE), limits of detection (LOD) and limits of quantification (LOQ).

**Analyte**	**Regression coefficients**	**Spiking level^a^**	**R_**E**_ ± RSD_**R**_ low level**	**R_**E**_ ± RSD_**R**_ intermediate level**	**R_**E**_ ± RSD_**R**_ high level**	**${\text{RSD}}_{\text{r}}^{\text{b}}$**	**SSE^c^**	**LOD**	**LOQ**
	***R*^**2**^**	**[ng/L]**		**[%]**	**[%]**	**[%]**	**[%]**	**[ng/L]**	**[ng/L]**
Aflatoxicol	0.9997	50/240/1200	91 ± 3	101 ± 4	96 ± 4	3/4/3	60	30	60
Aflatoxin B_1_	0.9995	10/48/240	96 ± 2	89 ± 4	84 ± 4	2/6/2	62	2.5	5.0
Aflatoxin B_2_	0.9997	20/48/240	100 ± 5	87 ± 6	84 ± 5	6/8/4	59	1.0	2.0
Aflatoxin G_1_	0.9997	30/48/240	99 ± 1	91 ± 7	88 ± 3	2/10/1	70	3.5	7.0
Aflatoxin G_2_	0.9996	30/48/240	100 ± 8	91 ± 8	90 ± 4	9/10/2	76	4.0	8.0
Aflatoxin M_1_	0.9997	10/48/240	109 ± 4	84 ± 5	92 ± 3	5/5/3	89	2.0	4.0
Aflatoxin M_2_	0.9994	48/100/240	87 ± 13	93 ± 8	88 ± 6	20/13/4	88	14	28
Aflatoxin P_1_	0.9995	48/80/240	91 ± 7	95 ± 6	89 ± 4	6/7/3	49	9.0	18
Aflatoxin Q_1_	0.9997	10/48/240	89 ± 8	91 ± 11	93 ± 4	6/13/2	75	13	26
Aflatoxin B_1_-N7-guanine	0.9997	20/240/1200	39 ± 19	22 ± 19	25 ± 25	26/20/38	90	4.0	8.0
Alternariol^d^	0.9996	50/96/480	-	-	6 ± 27	-/-/36	44	4.0	8.0
Alternariol monomethyl ether^e^	0.9998	10/96/480	86 ± 5	89 ± 4	86 ± 4	5/3/3	51	0.5	1.0
Beauvericin^e^	0.9993	6/10/48	85 ± 9	86 ± 6	87 ± 3	10/6/2	76	0.1	0.2
Citrinin	0.9998	6/48/240	118 ± 18	93 ± 4	88 ± 3	15/9/2	115	3.0	6.0
Deoxynivalenol^d^	0.9997	720/1000/3600	-	-	-	-/-/-	89	106	212
Dihydrocitrinone	0.9994	96/200/480	55 ± 7	41 ± 9	46 ± 15	4/14/17	114	14	28
Enniatin A	0.9997	6/10/48	99 ± 5	103 ± 6	99 ± 6	5/7/7	71	0.5	1.0
Enniatin A_1_	0.9994	6/10/48	98 ± 5	102 ± 8	97 ± 5	4/3/3	86	0.9	1.8
Enniatin B^e^	0.9997	6/10/48	85 ± 6	99 ± 11	87 ± 7	9/14/9	61	0.7	1.4
Enniatin ${\text{B}}_{1}^{\text{e}}$	0.9995	6/10/48	95 ± 6	100 ± 7	95 ± 5	4/7/4	71	0.5	1.0
HT-2 toxin	0.9966	720/2830/3600	84 ± 12	81 ± 9	98 ± 6	9/9/7	74	300	600
Nivalenol^d^	0.9998	800/1280/6400	-	-	-	-/-/-	91	70	140
Ochratoxin A^e^	0.9997	30/96/480	96 ± 3	109 ± 5	104 ± 5	4/3/3	80	0.8	1.5
Ochratoxin B	0.9997	20/96/480	97 ± 3	108 ± 5	105 ± 5	3/4/2	88	2.5	5.0
Ochratoxin α	0.9984	160/300/800	83 ± 18	75 ± 17	84 ± 4	24/23/8	93	24	48
Sterigmatocystin	0.9997	15/24/120	90 ± 2	86 ± 4	84 ± 4	2/3/2	34	0.5	1.0
T-2 toxin	0.9998	96/100/480	106 ± 5	95 ± 2	99 ± 5	6/2/4	55	11	22
Tentoxin	0.9995	96/200/480	101 ± 4	93 ± 3	101 ± 6	5/3/4	76	23	46
Zearalanone	0.9995	96/480/700	92 ± 4	89 ± 4	96 ± 2	3/3/1	50	60	120
α-Zearalanol	0.9996	128/640/800	103 ± 3	98 ± 5	97 ± 2	3/5/2	39	73	146
β-Zearalanol	0.9993	128/640/1200	98 ± 4	93 ± 5	95 ± 1	5/4/1	60	75	150
Zearalenone^e^	0.9997	96/100/480	103 ± 5	95 ± 3	98 ± 4	4/3/2	53	16	32
α-Zearalenol	0.9995	100/128/640	90 ± 5	103 ± 4	100 ± 5	5/5/4	48	44	87
β-Zearalenol	0.9997	100/128/640	92 ± 5	97 ± 6	93 ± 3	5/5/3	46	54	108

a *Spiking levels reported in the following order: low level/ intermediate level/ high level*.

b *RSD_r_ values reported in the following order: low level/ intermediate level/ high level*.

c *SSE calculated as the slope of calibration in matrix divided by the slope of calibration in solution expressed in percent*.

d *AOH, DON and NIV could not be recovered following our extraction procedure with the exception of AOH at the highest spiked level. Therefore, none of these toxins were successfully validated*.

e *Non-spiked pooled matrix sample was contaminated. Therefore, validation results reported were evaluated by standard addition*.

**Table 3:** Comparison of limit of detection (LOD) values for all analytes using the published (Braun et al., 2018) and newly presented approach.

	**LOD (ng/L)**		
**Sample preparation**	**QuEChERS^a^**	**QuEChERS + SPE**	**QuEChERS + SPE**
**LC-MS instrument**	**TSQ Vantage^a^**	**TSQ Vantage**	**QTrap6500^**+**^**
Aflatoxicol	150	75	30
Aflatoxin B1	40	10	2.5
Aflatoxin B2	42	8	1
Aflatoxin G1	43	7	3.5
Aflatoxin G2	79	18	4
Aflatoxin M1	43	5	2
Aflatoxin M2	76	8	14
Aflatoxin P1	68	22	9
Aflatoxin Q1	63	20	13
Aflatoxin B1-N7-guanine	200	40	4
Alternariol	-	10	4
Alternariol monomethyl ether	-	5	0.5
Beauvericin	6	0.5	0.1
Citrinin	25	3	3
Deoxynivalenol	770	225	106
Dihydrocitrinone	92	20	14
Enniatin A	5	2	0.5
Enniatin A1	12	2	0.9
Enniatin B	4	1	0.7
Enniatin B1	6	2	0.5
HT-2 toxin	1,400	455	300
Nivalenol	254	400	70
Ochratoxin A	48	5	0.8
Ochratoxin B	63	6	2.5
Ochratoxin α	210	34	24
Sterigmatocystin	13	2	0.5
T-2 toxin	180	33	11
Tentoxin	-	20	23
Zearalanone	-	76	60
α-Zearalanol	-	66	73
β-Zearalanol	-	50	75
Zearalenone	93	28	16

a *According to Braun et al. (2018)*.

### Validation of the Enhanced Clean-Up Protocol

The methods' performance was validated in-house according to established guidelines from EuraChem (Magnusson and Örnemark, 2014) and the European Commission Decision 2002/657/EC (European Commission Decision, 2002) by evaluating sensitivity, selectivity, linearity, repeatability, intermediate precision, extraction recovery and matrix effects. Overall, the validation was successful and the detailed results are reported in Table 2.

The enhanced method enabled the quantification of mycotoxins in the pg/L—ng/L range. Compared to our previously published method (Braun et al., 2018), LOD and LOQ values were decreased between a factor of two (α-ZEL) to 60x (BEA, OTA) depending on the analyte and ranged from 0.1 to 300 ng/L and 0.2 to 600 ng/L, respectively. Significant improvement in sensitivity was observed for most aflatoxins and ochratoxins with an improvement factor of ~20x (Table 3). In agreement with our previous publication, highest sample LOD values were observed for the polar trichothecenes DON, NIV and HT-2 (106, 70, and 300 ng/L) on both instruments. Selectivity of the method was assessed by evaluating a non-spiked pooled matrix extract in comparison to extracted spike samples. Selectivity was in concordance with the established guideline, if no co-eluting peak with a S/N ratio greater than three was found (European Commission Decision, 2002). For identification and quantification of spiked samples, parameters including retention time, parent and product ion as well as their ion ratio were evaluated. Here, the ion ratio proved to be reproducible over the concentration range tested (Table 2). Linearity of the instrument was assessed by weighted regression analysis (1/x) of concentrations tested within the matrix-matched calibration curve. Regression coefficients ranging from 0.9978 to 0.9998 demonstrated excellent linearity. Extraction recoveries were in good agreement with guideline requirements, except impaired extraction rates for AOH, AFB_1_-N7-guanine and DH-CIT (<6, <39, and <55%) and nearly no recovery for DON and NIV. Most of these exceptions can be reasonably explained by their polar character. While these analytes were not extractable from breast milk using acidified ACN, this is not the case for DH-CIT. Good extraction rates (mean R_E_: 100%) using the QuEChERS extraction only (Braun et al., 2018) suggest that this toxin is likely not fully retained by the SPE material. All other 29 analytes met the validation acceptance criteria with extraction recoveries ranging from 75 to 109% for all spiking levels, including the newly implemented analytes AME, TEN, ZAN, α-ZAL, and β-ZAL. Repeatability (RSD_r_) and intermediate precision (RSD_R_) for successfully validated analytes were mostly in the range of 2% to 9% with a maximum in RSD_r_ and RSD_R_ of 24 and 18% for e.g., CIT, HT-2 or OTα. These values are in good agreement for the spiked concentrations (mostly lower than 1000 ng/L). The EC guideline recommends to keep the RSD as low as possible for the extraction of analytes which were spiked at a concentration of 1000 ng/L or lower (European Commission Decision, 2002). No significant differences between RSD_R_ and RSD_r_ were observed. Matrix effects were assessed by comparing matrix-matched calibration slopes to solvent calibration slopes and this value was expressed in percent. Thus, a value above 100% indicates signal enhancement due to matrix effects and a value lower 100% indicates signal suppression. In addition, ^13^C-labeled reference standards were included to compensate for any diminished ionization of analytes and enhances the accuracy of these analytes. As expected, the enrichment of the matrix during sample clean-up resulted in a suppression of nearly all analytes. Highest matrix suppression was observed for STC with 34%. Typical characteristics could be observed depending on the chemical property of the analyte. For example, matrix effects for the group of ZEN, ZAN and their metabolites ranged from 39 to 60% and were thus more affected by the matrix than aflatoxins (59 to 89%). In contrast, CIT and DH-CIT with 114 and 115% exhibited slight signal enhancement, which is in concordance with our experience (Braun et al., 2018). Interestingly, matrix effects observed were comparable between instruments and, with the exception of CIT and DH-CIT, did not differ significantly.

### Limitations

The development of a broad multi-analyte method targeting highly diverse chemical classes is a difficult task. The selection of an appropriate clean-up strategy is complex, as the extraction may be either not possible without the loss of targeted analytes or the co-extraction of interfering matrix compounds. The toxins DON, NIV, AFB_1_-N7-guanine and AOH were not sufficiently recovered using our clean-up approach. Therefore, these analytes did not fulfill all required validation parameters. The extraction efficiency of DH-CIT was on average slightly lower than the requested validation criteria of 50% (European Commission Decision, 2002). However, this method can be used as a screening tool for DH-CIT, as the RSD for all evaluated spike samples was low (<15%). Fumonisins and tenuazonic acid were included in the method development, however, poor performance characteristics and decreased signal intensities or broad peaks and peak tailing impaired appropriate quantification.

### Proof-of-Principle Application of the Integrated QuEChERS-SPE Protocol

**Figure 3:**
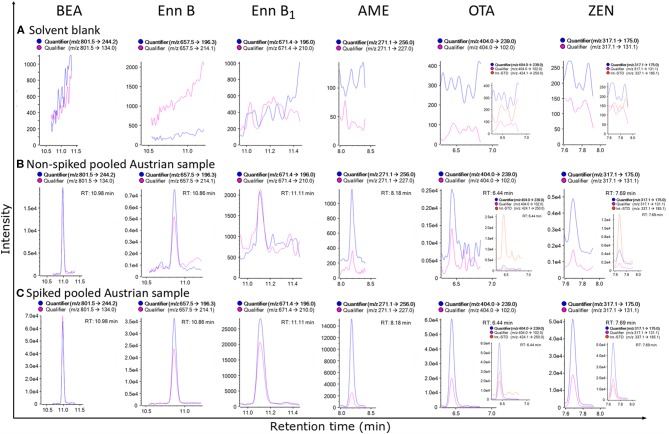
MRM-chromatograms of a solvent blank **(A)**, non-spiked pooled matrix from Austria, demonstrating the extremely high sensitivity of the established method, as this sample was considered a “blank” sample before **(B)** and spiked pooled matrix from Austria **(C)** of beauvericin (BEA), enniatin B (Enn B), enniatin B_1_ (Enn B_1_), alternariol monomethyl ether (AME), ochratoxin A (OTA) and zearalenone (ZEN), respectively. For OTA and ZEN ^13^C-labeled internal reference standards were included for analyte confirmation, while for the other analytes detected no labeled standards were available (To discriminate between quantifier and qualifier ion traces kindly refer to the online version of this figure).

**Figure 4:**
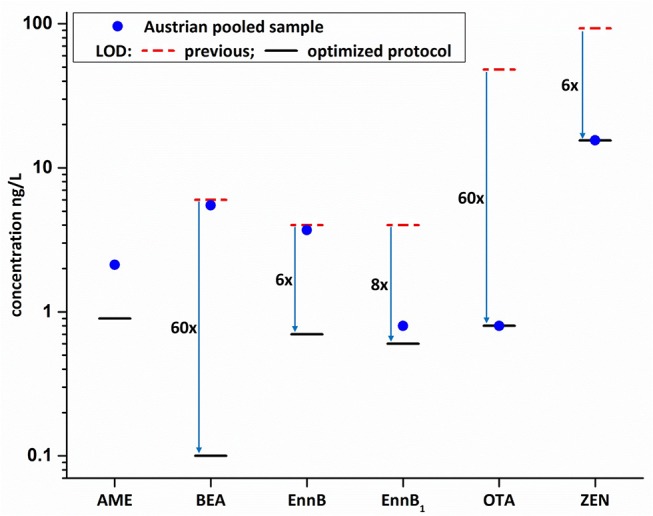
Comparison of previous methods' LOD values (Braun et al., 2018) with the LODs as obtained during in-house validation of the new methodology (blue arrows indicate sensitivity increase; AME was newly implemented within the present study). Mycotoxin concentration of the non-spiked pooled Austrian sample are indicated by the blue dot and highlight the feasibility to now detect and quantify chronic low-dose exposures.

The newly established protocol was applied to the pooled Austrian sample. This sample was initially considered as “blank matrix,” since it was not expected to detect mycotoxins in the pooled Austrian breast milk (Braun et al., 2018). However, the application of the enhanced assay resulted in the detection and quantification of several mycotoxins which demonstrates the significant increase in sensitivity of the newly established method. Generally, contamination of the pooled Austrian sample was low with the highest concentration of 6.2 ng/L found for BEA. Interestingly, the newly implemented *Alternaria* toxin AME was found in the pooled Austrian sample with a concentration of 2.1 ng/L (Figure 3). To the best of our knowledge, no data on AME in naturally contaminated breast milk samples has been published to date. Moreover, Enn B was quantified at a concentration of 4.7 ng/L. Other mycotoxins detected below their respective sample LOQ value included Enn B_1_, OTA and ZEN (Figure 4, Table S3). ZEN was recently shown to cross the placental barrier and exhibit synergistic toxic effects with other xenoestrogens (Vejdovszky et al., 2017a,b; Preindl et al., 2019; Warth et al., 2019). Hence, this finding needs to be confirmed in further surveys. Since only one pooled Austrian sample was available, no individual contamination patterns from Austrian volunteers could be assessed. This lack will be addressed in future large-scale biomonitoring studies.

**Figure 5:**
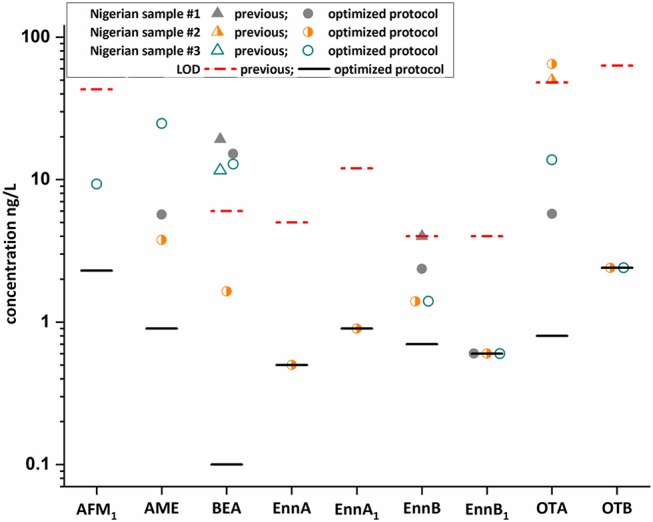
Comparison of three Nigerian samples using an old (Braun et al., 2018) and newly developed protocol. Results obtained after the integrated QuEChERS/SPE clean-up (colored dots) excellently fit with our previous reported concentration (colored triangles) for beauvericin (BEA), enniatin B (Enn B), and ochratoxin A (OTA) indicating the high precision of the developed method. In addition to the confirmed mycotoxins, six were newly identified and quantification was feasible for most mycotoxins. Importantly, aflatoxin M_1_ (AFM_1_) and OTA were confirmed by ^13^C-labeled reference standards.

Moreover, the optimized methodology was applied to a small set of randomly selected Nigerian samples and the results were compared with published data (Braun et al., 2018) (Figure 5, Table S3). The identification of AFM_1_, BEA, Enn B, and OTA was confirmed. However, the enhanced sample preparation protocol enabled the quantification of these analytes in the selected samples, which were mostly not detected or below the respective LOQ value when applying our previous approach. In addition, other mycotoxins, namely Enn A, Enn A_1_, Enn B_1_, OTB, and AME were identified. Interestingly, AME and OTA were the most abundant toxins in these samples with concentration up to 25 and 65 ng/L, respectively. The occurrence of mycotoxins in these breast milk samples can be reasonably explained, as most mycotoxins were found in household foods and plate-ready meals in Nigeria. Detected analytes included BEA with a frequency of 42 to 100% and concentration levels up to 435 μg/kg (Ezekiel et al., 2019; Ojuri et al., 2019). Moreover, AFM_1_ and OTA are recurring contaminants in food with levels up to 24 and 26 μg/kg and were also frequently found in Nigerian adult and infant urine with maximum levels of 620 and 310 ng/L (Šarkanj et al., 2018; Ezekiel et al., 2019; Ojuri et al., 2019). In breast milk samples, which were obtained from German and Chilean mothers (Munoz et al., 2010, 2013) particularly OTA was described as a frequent contaminant. While no multi-analyte method was suitable to quantify mycotoxin contamination in low exposure countries, the optimized assay with a LOD value of 0.8 ng/L for OTA demonstrated that our multi-analyte approach can compete even with tailored single-analyte methods (Munoz et al., 2013). Overall, the increased detection frequencies clearly demonstrate that the method is fit for purpose and can be applied to quantify trace levels of mycotoxins in breast milk. However, it has to be highlighted that the contamination levels detected are below any regulatory value in Europe (e.g., AFM_1_ below 25 ng/L) and therefore likely have no negative effect on infant health. Importantly, potential presence of mycotoxins in breast milk should not be a factor to avoid breastfeeding as the benefits clearly outweighs the risks and appropriate alternatives are mostly contaminated at higher levels. In addition, a recent study suggests that consumption of complementary infant food is unlikely to result in lower exposures (unpublished).

## Conclusion and Outlook

We report an optimized, highly sensitive and robust LC-MS/MS assay for the simultaneous quantification of mycotoxins and key metabolites in human breast milk. The rather polar mycotoxins AOH, DON, FBs, NIV, and AFB_1_-N7-Gua did not fulfill all required validation parameters and a more tailored approach has to be developed to assess these toxins. However, this is in line with our previous report in which the QuEChERS based extraction procedure was first optimized. For all other 29 analytes, the method proved to be reproducible down to the lower ng/L and even pg/L range. The improvement in sensitivity was achieved by elegantly linking QuEChERS and SPE extraction to enrich a broad range of chemically diverse toxicants. Importantly, the established unique sample preparation protocol might be employed in future large-scale investigations of environmental exposures within the exposome paradigm (Warth et al., 2017; Niedzwiecki et al., 2019). The developed protocol might be a solution for some of the analytical issues this emerging field is facing currently. Here, the methodological changes had a major impact on detection and quantification frequency as demonstrated in proof-of-principle measurements. In addition, several mycotoxins were found in a pooled Austrian sample demonstrating the ultimate sensitivity of this optimized approach. The detection of AME, which was not reported in this bio-fluid before, and the co-occurrence of mycotoxins highlight the need for large-scale epidemiological studies. Follow-on studies can be used to gain detailed insight into occurrence patterns, to estimate exposure of infants and to investigate potential correlations between exposure to mycotoxins and infant health effects. Overall, all these efforts are intended to minimize mycotoxin exposures in mothers and their infants throughout all critical life stages.

## Data Availability Statement

The datasets generated during and analyzed during the current study are available from the corresponding author on reasonable request.

## Ethics Statement

The studies involving human participants were reviewed and approved by the Ethic Committee of the University of Vienna (IRB#00157) and the Ethics Committee of Babcock University (#BUHREC294/16). The patients/participants provided their written informed consent to participate in this study.

## Author Contributions

DB conceived, designed and planned the experiments, performed LC-MS/MS measurements, data evaluation, interpretation and drafted the paper. CE conceived the experimental design, collected samples and supported data evaluation. DM was involved in the experimental design and data interpretation. BW designed and supervised the study and supported analyses and data evaluation/interpretation. All authors contributed to manuscript writing.

## Conflict of Interest

The authors declare that the research was conducted in the absence of any commercial or financial relationships that could be construed as a potential conflict of interest.

## Abbreviations: General

ACN: acetonitrile
CE: collision energy
CXP: cell exit potential
DP: declustering potential
ESI: electrospray ionization
SM: supplementary material
IS: internal standard
MeOH: methanol
MRM: multiple reaction monitoring
PSA: poly-secondary amine
R^2^: regression coefficient
R_E_: extraction recovery
SPE: solid phase extraction
SSE: signal suppression or enhancement.

**Mycotoxins**
AFL: aflatoxicol
AFB_1_: aflatoxin B_1_
AFB_1_-N7-guanine: aflatoxin B_1_-N7-guanine
AFB_2_: aflatoxin B_2_
AFG_1_: aflatoxin G_1_
AFG_2_: aflatoxin G_2_
AFM_1_: aflatoxin M_1_
AFM_2_: aflatoxin M_2_
AFP_1_: aflatoxin P_1_
AFQ_1_: aflatoxin Q_1_
AOH: alternariol
AME: alternariol monomethyl ether
BEA: beauvericin
CIT: citrinin
DH-CIT: dihydrocitrinone
DON: deoxynivalenol
Enn A: enniatin A
Enn A_1_: enniatin A_1_
Enn B: enniatin B
Enn B_1_: enniatin B_1_
FB_1_: fumonisin B_1_
FB_2_: fumonisin B_2_
HT-2: HT-2-toxin
NIV: nivalenol
OTA: ochratoxin A
OTB: ochratoxin B
OTα: ochratoxin alpha
STC: sterigmatocystin
T-2: T-2-toxin
TEN: tentoxin
ZAN: zearalanone
α-ZAL: alpha zearalanol
β-ZAL: beta zearalanol
ZEN: zearalenone
α-ZEL: alpha zearalenol
β-ZEL: beta zearalenol.
