# Supplementary material for: Exposure to Mycotoxin-Mixtures via Breast Milk: An Ultra-Sensitive LC-MS/MS Biomonitoring Approach
Source: Front Chem. 2020 May 19;8:423. doi: 10.3389/fchem.2020.00423 (PMC7248376; doi:10.3389/fchem.2020.00423)
Supplement: Supplementary file 1 [file Table_1.docx]

# Supplementary Material

**Exposure to mycotoxin-mixtures via breast milk:
An ultra-sensitive LC-MS/MS biomonitoring approach**

Dominik Braun^1^, Chibundu N. Ezekiel^2^, Doris Marko^1^, Benedikt Warth^1^*

^1^ University of Vienna, Faculty of Chemistry, Department of Food Chemistry and Toxicology, Vienna, Austria

^2^ Department of Microbiology, Babcock University, Ilishan Remo, Ogun State, Nigeria

Correspondence:

*Benedikt Warth, University of Vienna, Faculty of Chemistry, Department of Food Chemistry and Toxicology, Währinger Straße 38, 1090 Vienna, Austria.

Phone: +43 1 4277 70806

E-mail: [benedikt.warth@univie.ac.at](mailto:benedikt.warth@univie.ac.at)

## Supporting Materials and Methods

### MS and MS/MS parameters

Analytes on the QTrap®6500^+^ instrument were optimized using the ‘compound optimization’ tool included in the Analyst software. Hence, preferential parameters for declustering potential (DP), collision energy (CE) and cell exit potential (CXP) were determined for all analytes.

Operation parameters of the Turbo-V ion source were as follows: source temperature 450°C, curtain gas 30 psi, collision gas high, ion source gases (sheath and drying gas) 80 psi and the ion spray voltage was set to 4500 V in positive and -4500 V in negative mode.

## Supplementary Figures and Tables

### Supplementary Figure

Supplementary Figure S1. Solid phase extraction using a water preloaded reservoir to dilute the QuEChERS extract directly to 5% ACN


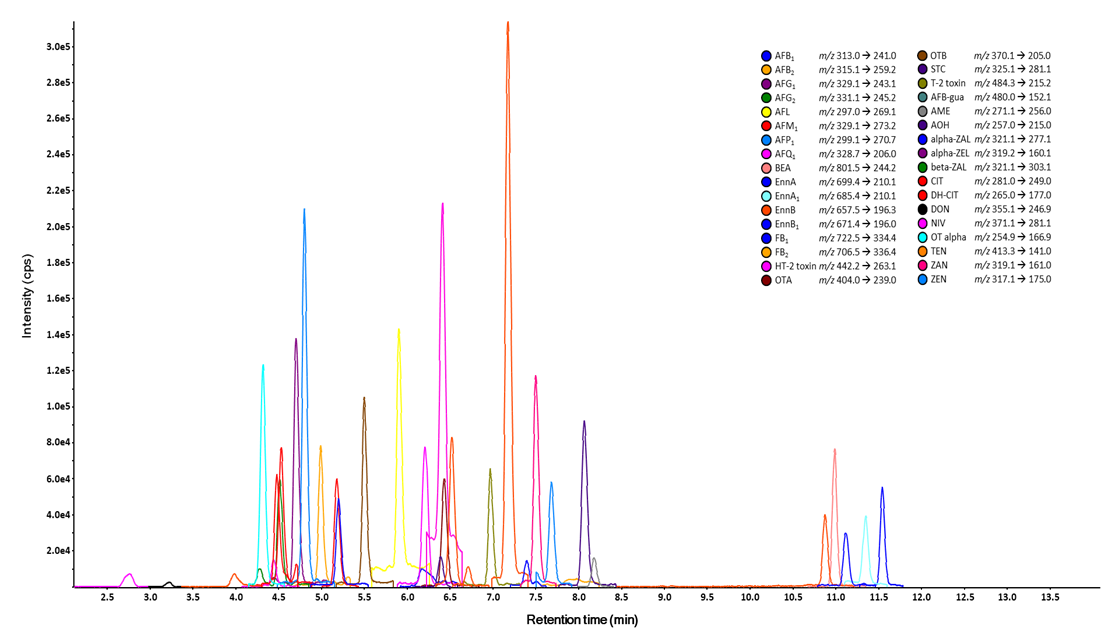


**Supplementary Figure S2.** MRM-chromatogram of a calibration standard in breast milk including all measured analytes


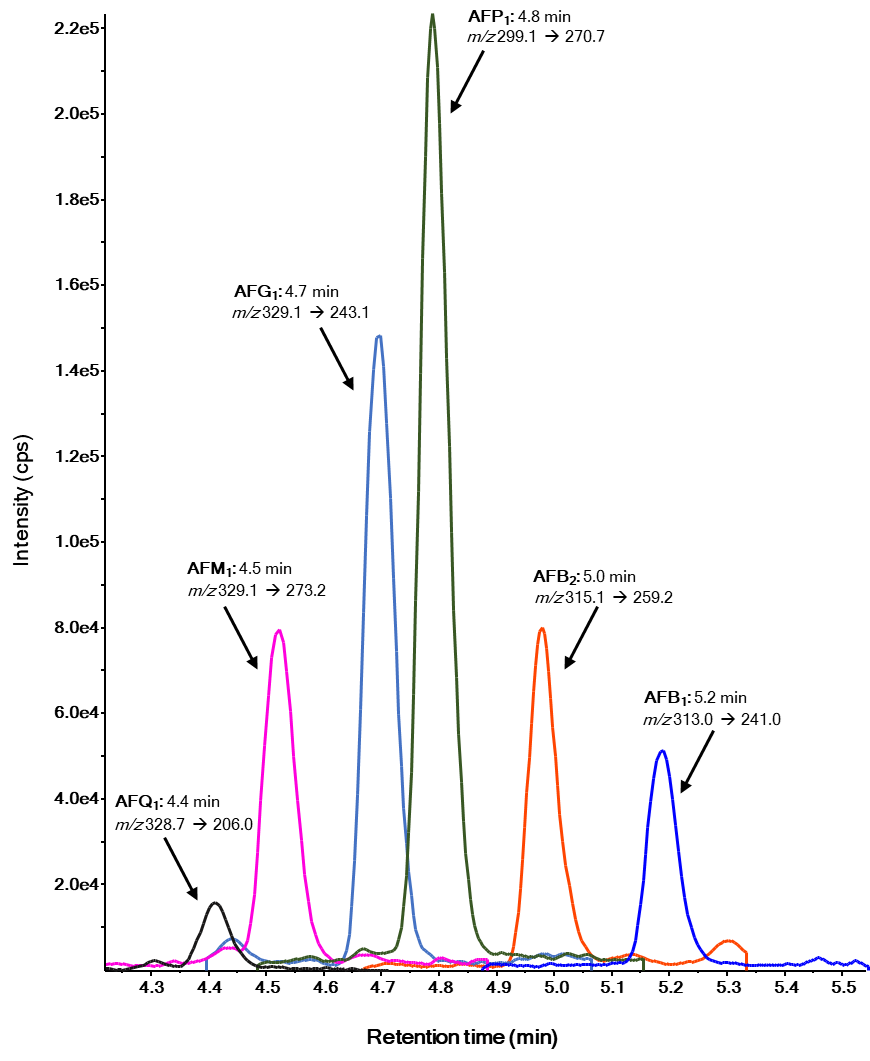


**Supplementary Figure S3.** Extracted MRM-chromatogram of an artificially spiked breast milk sample of selected aflatoxins

### Supplementary Tables

**Supplementary Table S1.** Analyte specific MS and MS/MS parameters as optimized on the TSQ Vantage instrument

| Analyte | t_R_^a^ | Precursor ion | Ion species | Product ion^b^ | CE^c^ | S-Lens | Dwell time^d^ |
| --- | --- | --- | --- | --- | --- | --- | --- |
|  | (min) | (*m/z*) |  | (*m/z*) | (V) | (eV) | (ms) |
| Aflatoxicol | 6.8 | 297.1 | [M-H_2_O+H]^+^ | 269.1/141.1 | 19/49 | 108 | 15/5 |
| Aflatoxin B_1_ | 6.1 | 313.1 | [M+H]^+^ | 285.1/128.1 | 22/69 | 122 | 15/5 |
| Aflatoxin B_2_ | 5.9 | 315.1 | [M+H]^+^ | 287.1/259.1 | 26/29 | 128 | 15/5 |
| Aflatoxin G_1_ | 5.6 | 329.1 | [M+H]^+^ | 243.1/200.0 | 26/40 | 105 | 15/5 |
| Aflatoxin G_2_ | 5.4 | 331.1 | [M+H]^+^ | 189.1/245.1 | 39/30 | 120 | 15/5 |
| Aflatoxin M_1_ | 5.4 | 329.1 | [M+H]^+^ | 273.0/229.0 | 24/41 | 126 | 15/5 |
| ^13^C-Aflatoxin M_1_ | 5.4 | 346.1 | [M+H]^+^ | 288.2 | 24 | 126 | 15 |
| Aflatoxin M_2_ | 5.2 | 331.1 | [M+H]^+^ | 273.0/285.0 | 24/23 | 121 | 15/5 |
| Aflatoxin P_1_ | 5.7 | 299.0 | [M+H]^+^ | 271.0/215.0 | 24/28 | 136 | 15/5 |
| Aflatoxin Q_1_ | 5.3 | 329.1 | [M+H]^+^ | 206.0/177.0 | 24/34 | 126 | 15/5 |
| Aflatoxin B_1_-N7-guanine | 4.8 | 480.2 | [M+H]^+^ | 152.0/135.0 | 20/50 | 85 | 15/5 |
| Alternariol | 7.2 | 257.1 | [M-H]^-^ | 215.1/147.1 | 27/33 | 70 | 15/5 |
| ^2^H-Alternariol | 7.2 | 261.0 | [M-H]^-^ | 150.0 | 27 | 70 | 15 |
| Alternariol monomethyl ether | 9.0 | 271.1 | [M-H]^-^ | 256.1/227.1 | 23/38 | 73 | 15/5 |
| Beauvericin | 11.9 | 801.4 | [M+NH_4_]^+^ | 243.9/133.9 | 29/51 | 138 | 15/5 |
| Citrinin | 6.1 | 281.1 | [M+MeOH-H]^-^ | 205.0/175.0 | 24/39 | 85 | 15/5 |
| ^13^C-Citrinin | 6.1 | 294.1 | [M+MeOH-H]^-^ | 262.0 | 24 | 85 | 15 |
| Deoxynivalenol | 4.0 | 355.1 | [M+OAc]^-^ | 265.2/247.2/59.0 | 17/19/19 | 75 | 15/5/5 |
| ^13^C-Deoxynivalenol | 4.0 | 370.1 | [M+OAc]^-^ | 278.8 | 17 | 75 | 15 |
| Dihydrocitrinone | 5.4 | 265.1 | [M-H]^-^ | 221.1/247.1 | 20/19 | 71 | 15/5 |
| Enniatin A | 12.6 | 699.5 | [M+NH_4_]^+^ | 210.0/228.0 | 29/29 | 127 | 15/5 |
| Enniatin A_1_ | 12.3 | 685.5 | [M+NH_4_]^+^ | 210.1/228.2 | 29/28 | 113 | 15/5 |
| Enniatin B | 11.8 | 657.4 | [M+NH_4_]^+^ | 196.3/214.0 | 28/31 | 114 | 15/5 |
| Enniatin B_1_ | 12.0 | 671.4 | [M+NH_4_]^+^ | 196.0/210.0 | 29/29 | 114 | 15/5 |
| HT-2 toxin | 7.1 | 442.2 | [M+NH_4_]^+^ | 263.1/215.0 | 12/12 | 68 | 15/5 |
| Nivalenol | 3.5 | 371.1 | [M+OAc]^-^ | 281.1/311.2/59.1 | 22/11/19 | 66 | 15/5/5 |
| ^13^C-Nivalenol | 3.5 | 386.1 | [M+OAc]^-^ | 295.2 | 22 | 66 | 15 |
| Ochratoxin A | 7.4 | 404.1 | [M+H]^+^ | 239.0/102.0 | 25/62 | 99 | 15/5 |
| ^13^C-Ochratoxin A | 7.4 | 424.1 | [M+H]^+^ | 250.0 | 25 | 99 | 15 |
| Ochratoxin B | 6.5 | 370.1 | [M+H]^+^ | 205.0/103.0 | 21/49 | 84 | 15/5 |
| Ochratoxin α | 5.2 | 254.9 | [M-H]^-^ | 166.9/210.9 | 25/17 | 74 | 15/5 |
| Sterigmatocystin | 8.9 | 325.1 | [M+H]^+^ | 281.1/310.1 | 37/24 | 118 | 15/5 |
| T-2 toxin | 7.9 | 484.3 | [M+NH_4_]^+^ | 215.1/185.1 | 14/20 | 83 | 15/5 |
| Tentoxin | 7.4 | 413.2 | [M-H]^-^ | 271.2/141.1 | 19/22 | 100 | 15/5 |
| Zearalanone | 8.3 | 319.2 | [M-H]^-^ | 275.2/205.2 | 23/23 | 115 | 15/5 |
| α-Zearalanol | 8.0 | 321.2 | [M-H]^-^ | 277.2 | 23/24 | 110 | 15/5 |
| β-Zearalanol | 7.3 | 321.2 | [M-H]^-^ | 277.2/303.3 | 23/24 | 110 | 15/5 |
| Zearalenone | 8.5 | 317.1 | [M-H]^-^ | 175.1/131.1 | 25/30 | 123 | 15/5 |
| ^13^C-Zearalenone | 8.5 | 335.1 | [M-H]^-^ | 185.1 | 25 | 123 | 15 |
| α-Zearalenol | 8.2 | 319.1 | [M-H]^-^ | 275.3/160.1 | 22/29 | 82 | 15/5 |
| β-Zearalenol | 7.6 | 319.0 | [M-H]^-^ | 275.3/160.0 | 22/33 | 82 | 15/5 |

^a^ Retention time.

^b^ Quantifier/qualifier.

^c^ Collision energy.

^d^ Reported as quantifier/qualifier dwell time.

**Supplementary Table S2**. Performance characteristics of the method as obtained during in-house validation on the TSQ Vantage instrument including concentration range of matrix matched standard calibration, regression coefficient (R^2^), spiking levels, recoveries of the extraction step (R_E_), intermediate precision (RSD_R_), repeatability (RSD_r_), signal suppression/enhancement (SSE), limits of detection (LOD) and limits of quantification (LOQ).

| Analyte | Regression coefficients | Spiking level^a^ | R_E_ ± RSD_R_  intermediate level | R_E_ ± RSD_R_  high level | RSD_r_^b^ | SSE^c^ | LOD | LOQ |
| --- | --- | --- | --- | --- | --- | --- | --- | --- |
|  | R² | [ng L^-1^] | [%] | [%] | [%] | [%] | [ng L^-1^] | [ng L^-1^] |
| Aflatoxicol | 0.9940 | 240/1200 | 83 ± 4 | 87 ± 2 | 4/2 | 56 | 75 | 150 |
| Aflatoxin B_1_ | 0.9841 | 48/240 | 85 ± 10 | 86 ± 2 | 11/3 | 69 | 10 | 20 |
| Aflatoxin B_2_ | 0.9889 | 48/240 | 87 ± 12 | 86 ± 9 | 18/7 | 72 | 8.0 | 16 |
| Aflatoxin G_1_ | 0.9894 | 48/240 | 79 ± 12 | 90 ± 8 | 8/5 | 71 | 7.0 | 14 |
| Aflatoxin G_2_ | 0.9826 | 48/240 | 76 ± 26 | 81 ± 8 | 24/9 | 80 | 18 | 36 |
| Aflatoxin M_1_ | 0.9929 | 48/240 | 71 ± 15 | 85 ± 7 | 11/5 | 94 | 5.0 | 10 |
| Aflatoxin M_2_ | 0.9697 | 48/240 | 74 ± 14 | 76 ± 11 | 9/14 | 90 | 8.0 | 16 |
| Aflatoxin P_1_ | 0.9808 | 48/240 | 79 ± 15 | 79 ± 10 | 7/10 | 65 | 22 | 44 |
| Aflatoxin Q_1_ | 0.9817 | 48/240 | 71 ± 17 | 83 ± 6 | 17/4 | 82 | 20 | 40 |
| Aflatoxin B_1_-N7-guanine | 0.9719 | 240/1200 | 26 ± 42 | 29 ± 31 | 18/41 | 94 | 40 | 80 |
| Alternariol^d^ | 0.9800 | 96/480 | 4 ± 44 | 3 ± 33 | 53/31 | 38 | 10 | 20 |
| Alternariol monomethyl ether^e^ | 0.9948 | 96/480 | 71 ± 6 | 73 ± 3 | 7/5 | 43 | 5.0 | 10 |
| Beauvericin^e^ | 0.9901 | 10/48 | 91 ± 9 | 86 ± 6 | 7/9 | 62 | 0.5 | 1.6 |
| Citrinin | 0.9965 | 48/240 | 117 ± 22 | 95 ± 21 | 14/26 | 85 | 3.0 | 6.0 |
| Deoxynivalenol^d^ | 0.9625 | 720/3600 | - | - | - | 83 | 225 | 450 |
| Dihydrocitrinone | 0.9866 | 96/480 | 36 ± 28 | 34 ± 32 | 26/35 | 83 | 20 | 40 |
| Enniatin A | 0.9913 | 9.6/48 | 67 ± 15 | 79 ± 5 | 9/7 | 65 | 2.0 | 4.0 |
| Enniatin A_1_ | 0.9741 | 9.6/48 | 54 ± 24 | 95 ± 26 | 25/17 | 71 | 2.0 | 4.0 |
| Enniatin B^e^ | 0.9766 | 9.6/48 | 73 ± 36 | 83 ± 12 | 23/8 | 51 | 1.0 | 2.0 |
| Enniatin B_1_^e^ | 0.9612 | 9.6/48 | 55 ± 34 | 78 ± 9 | 15/8 | 63 | 2.0 | 4.0 |
| HT-2 toxin | 0.9877 | 720/3600 | 64 ± 43 | 102 ± 10 | 31/7 | 90 | 455 | 910 |
| Nivalenol^d^ | 0.9979 | 1280/6400 | - | - | - | 97 | 400 | 800 |
| Ochratoxin A^e^ | 0.9890 | 96/480 | 77 ± 5 | 88 ± 5 | 5/5 | 86 | 5.0 | 10 |
| Ochratoxin B | 0.9941 | 96/480 | 89 ± 5 | 93 ± 4 | 5/4 | 87 | 6.0 | 12 |
| Ochratoxin α | 0.9865 | 160/800 | 62 ± 24 | 60 ± 9 | 14/10 | 83 | 34 | 68 |
| Sterigmatocystin | 0.9797 | 24/120 | 78 ± 20 | 87 ± 4 | 15/6 | 57 | 2.0 | 4.0 |
| T-2 toxin | 0.9845 | 96/480 | 81 ± 17 | 89 ± 10 | 29/13 | 70 | 33 | 66 |
| Tentoxin | 0.9936 | 96/480 | 78 ± 13 | 83 ± 6 | 11/6 | 74 | 20 | 40 |
| Zearalanone | 0.9592 | 96/480 | 71 ± 45 | 81 ± 16 | 72/11 | 40 | 76 | 152 |
| α-Zearalanol | 0.9768 | 128/640 | 77 ± 12 | 83 ± 3 | 12/5 | 32 | 66 | 132 |
| β-Zearalanol | 0.9931 | 128/640 | 78 ± 10 | 76 ± 5 | 7/4 | 55 | 50 | 100 |
| Zearalenone | 0.9861 | 96/480 | 87 ± 11 | 75 ± 8 | 7/5 | 45 | 28 | 56 |
| α-Zearalenol | 0.9265 | 128/640 | 46 ± 85 | 89 ± 10 | 30/17 | 38 | 58 | 116 |
| β-Zearalenol | 0.9833 | 128/640 | 75 ± 22 | 77 ± 5 | 19/5 | 51 | 64 | 128 |

^a^ Spiking levels reported in the following order: intermediate level/ high level.

^b^ RSD_r_ values reported in the following order: intermediate level/ high level.

^c^ SSE calculated as the slope of calibration in matrix divided by the slope of calibration in solution expressed in percent.

^d^ AOH, DON and NIV could not be recovered following our extraction procedure with the exception of AOH at the highest spiked level. Therefore, none of these toxins were successfully validated.

^e^ Non-spiked pooled matrix sample was contaminated. Therefore, validation results reported were evaluated by standard addition.

**Supplementary Table S3.** Concentration levels of mycotoxins found in the pooled Austrian sample and a small set of Nigerian samples (n=3) using the optimized protocol. Individual Nigerian sample concentration was compared with data obtained following our previous protocol.

|  | **AFM_1_** | **AME** | **BEA** | **EnnA** | **EnnA_1_** | **EnnB** | **EnnB_1_** | **OTA** | **OTB** | **ZEN** |
| --- | --- | --- | --- | --- | --- | --- | --- | --- | --- | --- |
|  | **ng L^-1^** |  |  |  |  |  |  |  |  |  |
| LOD | 2.0 | 0.5 | 0.1 | 0.5 | 0.9 | 0.7 | 0.5 | 0.8 | 2.5 | 16 |
| Austrian pooled sample | n.d | 1.9 | 5.4 | n.d | n.d | 3.7 | <LOQ | <LOQ | n.d | <LOQ |
| Nigerian sample #1 | n.d | 5.7 | 15 | n.d | n.d | 2.4 | <LOQ | 5.7 | n.d | n.d |
| Nigerian sample #2 | n.d | 3.8 | 1.6 | <LOQ | <LOQ | <LOQ | <LOQ | 65 | LOD | n.d |
| Nigerian sample #3 | 5.3 | 25 | 13 | n.d | n.d | <LOQ | <LOQ | 14 | LOD | n.d |
|  |  |  |  |  |  |  |  |  |  |  |
| **Previous protocol**  **(Braun *et al*. 2018 )** |  |  |  |  |  |  |  |  |  |  |
| LOD | 43 | n.a | 6.0 | 5.0 | 12 | 4.0 | 4.0 | 48 | 63 | 93 |
| Austrian pooled sample | n.d | n.a | n.d | n.d | n.d | n.d | n.d | n.d | n.d | n.d |
| Nigerian sample #1 | n.d | n.a | 19 | n.d | n.d | <LOQ | n.d | n.d | n.d | n.d |
| Nigerian sample #2 | n.d | n.a | n.d | n.d | n.d | n.d | n.d | <LOQ | n.d | n.d |
| Nigerian sample #3 | n.d | n.a | 12 | n.d | n.d | n.d | n.d | n.d | n.d | n.d |

LOD: limit of detection.

LOQ: limit of quantification.

n.d.: not detected.

n.a.: not assessed.

## References

Braun, D.; Ezekiel, C.N.; Abia, W.A.; Wisgrill, L.; Degen, G.H.; Turner, P.C.; Marko, D.; Warth, B. Monitoring Early Life Mycotoxin Exposures via LC-MS/MS Breast Milk Analysis. Anal Chem 2018;90:14569-14577.
